# Pilot study of facial and bodily feedback

**DOI:** 10.1007/s40211-022-00426-z

**Published:** 2022-08-26

**Authors:** Chloë Hutchings-Hay, Marcela M. Dapelo, Gisselle Briceño, Camila Fernández, Kate Tchanturia

**Affiliations:** 1 Department of Psychological Medicine, King’s College London, London, UK; 2 Eating Behaviour Program, Mental Health Service, Clinica Universidad de los Andes, Santiago, Chile; 3 School of Psychology, Universidad Adolfo Ibáñez, Santiago, Chile; 4 Psychological Set Research and Correction Center, Tbilisi State Medical University, Tbilisi, Georgia

**Keywords:** Facial feedback hypothesis, Modulation, Smile, Bodily posture, Positive affect, Gesichtsfeedback, Modulation, Lächeln, Körperhaltung, Positiver Affekt

## Abstract

**Background:**

The modulation hypothesis of facial feedback has not adequately examined how combining facial expressions and bodily postures might influence our experience of emotional stimuli. This pilot study examined a new method for manipulating both face and body together, which is important in furthering our understanding of how face and body interact to influence emotional experiences in the real world.

**Methods:**

Using a within-subjects design, 30 participants viewed positive film clips under four conditions: (1) positive face with positive body (PP), (2) positive face with neutral body (PN), (3) neutral face with positive body (NP) and (4) neutral face with neutral body (NN). Measures of positive and negative affect were taken before and after each clip, to assess the subjective emotional experience.

**Results:**

Repeated-measures analysis of variance (ANOVA) was conducted to examine differences in the emotional experience under each condition. Post hoc pairwise comparisons demonstrated that positive affect in the PP condition was significantly higher than in the NP and NN conditions. There was no significant difference between the PP and NN conditions.

**Conclusion:**

Whilst the study findings are difficult to interpret, this pilot study generated a number of important methodological learnings that are relevant to future research of this kind.

## Abstract (de)

**Hintergrund:**

Die Modulationshypothese des Gesichtsfeedbacks hat nicht ausreichend untersucht, wie die Kombination von Gesichtsausdruck und Körperhaltung unser Erleben von emotionalen Reizen beeinflussen könnte. Diese Pilotstudie untersuchte eine neue Methode zur gleichzeitigen Manipulation von Gesicht und Körper, die wichtig ist, um zu verstehen, wie Gesicht und Körper interagieren, um emotionale Erfahrungen in der realen Welt zu beeinflussen.

**Methodik:**

Insgesamt 30 Teilnehmende sahen sich positive Filmclips unter vier Bedingungen an: (1) positives Gesicht mit positivem Körper (PP), (2) positives Gesicht mit neutralem Körper (PN), (3) neutrales Gesicht mit positivem Körper (NP) und (4) neutrales Gesicht mit neutralem Körper (NN). Vor und nach jedem Clip wurde der positive und negative Affekt gemessen, um die subjektive emotionale Erfahrung zu bewerten.

**Ergebnisse:**

Eine Varianzanalyse mit wiederholten Messungen (ANOVA) wurde durchgeführt, um die Unterschiede im emotionalen Erleben unter jeder Bedingung zu untersuchen. Post-hoc-Paarvergleiche zeigten, dass der positive Affekt in der PP-Bedingung signifikant höher war als in den NP- und NN-Bedingungen. Es gab keinen signifikanten Unterschied zwischen der PP- und der NN-Bedingung.

**Schlussfolgerung:**

Die Ergebnisse der Studie sind zwar schwer zu interpretieren, jedoch hat diese Pilotstudie eine Reihe wichtiger methodischer Erkenntnisse erbracht, die für künftige Forschungen dieser Art von Bedeutung sind.

## Introduction

The facial feedback hypothesis incorporates a group of hypotheses describing the effects of facial action on the emotional experience, both physiological and subjective. McIntosh [1] outlined four possible routes through which facial expressions influence the emotional experience: (1) facial configurations correspond to emotions, (2) facial movements modulate emotions, (3) facial actions initiate emotions, or (4) facial action is required for emotions.

The present study focuses on the second hypothesis proposed by McIntosh [1]: the modulation hypothesis (also referred to as the ‘monotonicity’ hypothesis by Soussignan [2]). Modulation refers to how, in the presence of external emotional stimuli, facial movements (or lack thereof) may increase (or decrease) how intensely an emotion is felt [1].

The modulation hypothesis was brought to attention by Strack and colleagues’ seminal study [3] wherein participants were asked to hold a pen using either only their lips (inhibiting a smile) or only their teeth (facilitating a smile) whilst rating the funniness of cartoons. The study showed that participants who held a pen with their teeth reported feeling more amused than those who held it with their lips. This was taken as evidence that smiling amplified the positive emotional experience. The study had great influence in the field and was followed by others using similar paradigms [2].

However, a failed replication [4] has called into question the validity of this finding. It has been suggested that a methodological difference might explain the lack of replication: awareness of video-recording. Noah and colleagues [5] suggest that feeling observed may reduce reliance on internal cues, reducing facial feedback effects. In fact, their study found evidence for facial feedback effects when participants were not being recorded, but a reduced effect when participants were aware of being monitored [5]. Covertly manipulating participants’ facial expressions into natural emotional displays, whilst keeping participants unaware of experimental aims, remains a key challenge within this field of research [6].

Methodologically speaking, Strack and colleagues’ [3] between-groups design may also have been inappropriate. It has been suggested that facial feedback effects should be measured within each individual [1]. This is because there can be wide variability between people in terms of emotional expressivity, which is shaped by numerous factors including gender and personality [7, 8].

Despite concerns about the validity of the ‘pen-in-mouth’ paradigm, a subsequent meta-analysis has confirmed a small but significant modulation effect for facial expressions in amplifying positive emotions [9]. Moreover, a forthcoming report from over 3800 participants across 19 countries has provided evidence that both a facial mimicry and a voluntary facial action task intensify feelings of happiness [10]. This therefore implies that the ‘pen-in-mouth’ paradigm may be the problem, rather than the modulation hypothesis in and of itself.

An important gap in the literature is understanding how the face works in conjunction with the body in modulating our emotional experience. Most of the literature has focused upon facial feedback. However, early theorists did not limit this feedback effect to facial action; they also considered the effect of bodily movements and postures [11].

Relevant to the present study, evidence points to the benefits of upright posture in initiating positive mood. For example, participants randomly assigned to sit upright reported higher self-esteem and better mood than those sitting slumped [12], and an upright posture was associated with reduced negative affect and anxiety in individuals with depression [13].

Less is known about how bodily postures can modulate our responses to emotional stimuli. One study showed that leaning forward (as opposed to reclining) increased responsiveness on electrophysiological measures when participants were viewing erotic stimuli [14]. Further, evidence suggests that an upright bodily posture (as opposed to a slumped posture) might be conducive to a more positive mind-set during stressful tasks [12].

Even less is understood about how the body and face together can modulate responses to emotional stimuli. One study investigating the independent feedback effect of facial expression and bodily postures found that facial expressions have a stronger impact than postures [15]. However, this study had participants display facial expressions and postures sequentially, which is not reflective of real life, where emotional expression is a combination of face and body together.

Flack and colleagues [16] tested the feedback effects of facial expression, bodily postures, and the face and body in combination across two studies. First, the authors evaluated whether participants reported higher levels of emotion when they were displaying the congruent combination of face and posture. The results confirmed that participants reported higher levels of anger when displaying an angry face and posture (compared to a sad, fearful, or happy face and posture combination). Similar findings were shown for sadness, fear and happiness. Secondly, the authors tested if the feedback effect for the combined facial expression and bodily posture was higher than either alone. The results showed that the combination of face and bodily postures of anger, sadness and fear was associated with significantly higher self-reported scores of anger, sadness, and fear, respectively, compared to face or posture alone. However, these findings did not hold for happiness and disgust.

Taken together, these findings provide preliminary support for a stronger feedback effect for the combination of an emotional facial and bodily display of emotion, as compared to either facial or bodily displays in isolation. However, the study only considered the combined feedback effect for congruent emotional displays. Moreover, part of the study involved instructing participants to either manipulate their face or their body. Therefore, the researchers have not controlled for participants adopting an incongruent face or bodily expression of emotion. These findings are limited because naturalistic emotional displays involve manipulating both at once, and incongruencies between the two might alter the emotional experience.

This pilot study aims to explore a new study design for manipulating both face and body simultaneously, which is important in furthering our understanding of how facial and bodily feedback work together. Study designs involving both face and body are important in making this area of research more relevant to naturalistic emotional displays. The present research takes into account prior limitations in the evidence base, by employing a within-subjects design, limiting and checking for demand characteristics and manipulating both the face and body simultaneously to understand how congruent and incongruent emotional displays may have an impact.

## Methods

### Participants

In all, 50 women participated in this study. However, one participant had to be excluded due to reporting a diagnosis of mental illness, two were excluded due to technical issues (a malfunction with the video-recording device), one had to be excluded because she guessed the study purpose, and 16 had to be excluded after a validity check (see ‘Validity check’ section). The final sample therefore consisted of 30 women from London (mean age 26.17, standard deviation [SD] 8.44). Men were not included because reviews of the evidence point to a reliable difference in outward emotional expressivity, with women being more emotionally expressive than men [7].

Sample size was calculated using G*Power. It was determined that a sample of 45 participants would be required to assess a medium effect size (Cohen’s d = 0.5) with 80% power, considering α = 0.05.

Inclusion criteria were being female, older than 18 years of age, and fluency in English. Additional exclusion criteria were the following: having a diagnosis of mental illness, having facial paralysis, and having ever used botulinum toxin A (Botox). In order to screen participants, a demographics questionnaire was completed by participants, including questions on whether or not participants had ever been diagnosed with a psychiatric disorder, whether or not they had facial paralysis or whether or not they had used Botox previously. The exclusion criteria were selected as these were deemed to be factors that could have influenced task compliance or facial and bodily feedback processes.

### Procedure

Participants were initially informed about the study procedures, but the real purpose of the study was concealed. The study was instead advertised as to do with gender differences in art perception. Deception was considered necessary because awareness of the study aim could have altered participants’ response in order to conform to experimental demands [1]. The study was approved by the King’s College London Ethics Committee (ref. HR/16/17-3744).

During the experimental task, participants were video-recorded. At the end of the task, the researcher assessed participants’ awareness of the study aims. Participants were then fully debriefed as to the true experimental aims and compensated for their participation with £ 10. Any questions or concerns were fully addressed during this debrief. No participant reported any concern with the deception used in this study, or the true experimental aims.

### Experimental task

**Fig. 1:**
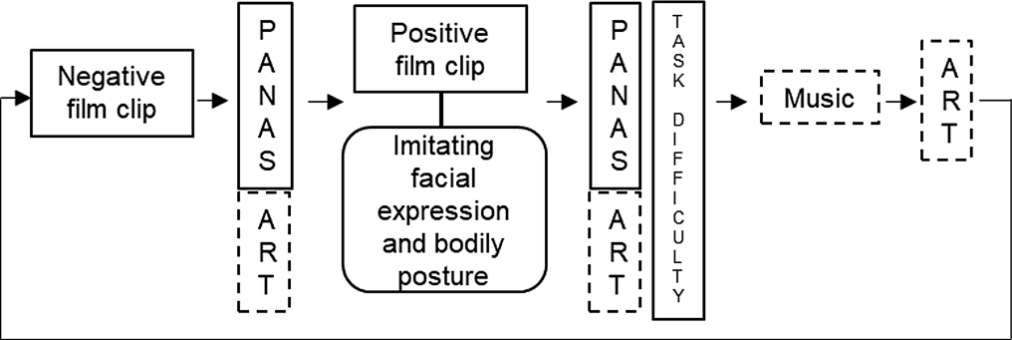
Diagram of the experimental task used in this study. The* solid line boxes* indicate key study measures. *Dotted line boxes* indicate the parts of the study concerning the cover story/filler tasks. *PANAS* Positive and Negative Affect Schedule, *ART* art interpretation task (filler task)

The experimental task involved participants firstly watching a negative film clip to induce negative affect. The Positive and Negative Affect Schedule (PANAS) [17] was then completed. Participants were shown photographs of facial expressions and bodily postures to hold whilst watching the next film clip. The next film clip was selected so as to induce positive affect. Participants then completed the PANAS again, as well as a measure of task difficulty. A filler task (a music clip was played and participants were asked to give their interpretation) was used between conditions to support the cover story (Fig. 1).

As the study employed a within-subjects design, every participant went through the above procedure four times during the study. Eight film clips were used in the study (four negative and four positive clips). The negative videos were taken from a validated set of films shown to elicit emotions [18]; the positive videos were selected based on a pilot study (data available upon request). The positive film clips ranged in duration from 2 min 4 s to 2 min 53 s. The order of these film clips was randomized. The order of the experimental conditions was also randomized. The four conditions each participant experienced were the following: (1) Neutral facial expression and neutral posture (NN); (2) Neutral facial expression and positive posture (NP); (3) Positive facial expression and neutral posture (PN), and (4) Positive facial expression and positive posture (PP) (Table 1).

**Table 1:** Facial expressions and bodily postures

Facial expression	Bodily posture	
	Neutral	Positive
Neutral	Neutral face + Slumped sitting = NN	Neutral face + Upright sitting = NP
Positive	Smiling face + Slumped sitting = PN	Smiling face + Upright sitting = PP

### Materials

#### Mood measurement

The Positive and Negative Affect Schedule (PANAS) [17] was used as the dependent variable to capture mood ratings. The PANAS is a widely used measure rating the extent to which participants have experienced 20 emotions, 10 of which are positive (e.g. ‘interested’) and 10 of which are negative (e.g. ‘scared’).

#### Facial and bodily manipulations

Five positive and five neutral facial expressions were taken from the Pictures of Facial Affect set [19] and used as stimuli for this study. In addition, a photograph of a woman sitting in a slumped posture, and a man sitting in an upright posture were used as the bodily posture stimuli.

### Manipulation checks

#### Validity check

Video-recordings were taken of participants during the experimental task. Research assistants, who were blind to the study design and purpose, viewed the recordings and rated the percentage of time each participant spent displaying a smile, a neutral expression, sitting upright or sitting in a slumped posture. Participants were excluded if they did not follow instructions as instructed for at least 75% of the time when watching each clip. Whilst all participants followed instructions regarding bodily postures, 16 participants did not follow instructions for facial expressions. All of these 16 participants failed to maintain a smile for the duration of the clip when instructed to do so, and of these participants 9 did not smile at all when instructed. As a result, all 16 participants had to be excluded because instructions had not been adequately followed.

Twenty videos selected at random were coded by two research assistants, and two-way random intraclass correlation (ICC) with absolute agreement was used to assess intercoder reliability, showing excellent agreement (ICC (2,2) = 1.00 for facial expression, and ICC (2,2) = 1.00 for bodily posture).

#### Task difficulty

After each of the four experimental conditions, participants rated task difficulty on a 5-point Likert scale (ranging from 1 = “Not at all difficult” to 5 = “Extremely difficult”).

### Statistical analysis

Descriptive statistics were used to initially explore the dataset. For the main analysis, the difference between self-reported affect rated before and after watching the positive film clip was calculated. This difference was calculated separately for positive and negative affect (using the positive and negative subscale of PANAS) and used as a measure of the affective experience (dependent variable). Repeated-measures analysis of variance (ANOVA) was used to evaluate the effect of each condition (NN, NP, PN, and PP) on the self-reported affective experience.

Distributions were explored using histograms and Shapiro–Wilk test. For positive affect, only one condition followed the normal distribution, while the other three were positively skewed. Thus, log transformation was used. To eliminate negative numbers and 0 a constant equal to the maximum negative number in the data plus one was added to all data [20]. Then, log transformation was conducted, yielding satisfactory results.

For negative emotions, two conditions followed the normal distribution (NN and PP), while the others were negatively skewed. Therefore, negative numbers and 0 were eliminated in the same fashion described for positive affect, and then scores were reversed by subtracting each score from the highest scored obtained plus one [20]. Finally, square root transformation was applied, with satisfactory results.

The transformed data was used for the main statistical analysis. Two repeated-measures ANOVAs were conducted (one for positive affect and one for negative affect) in which each condition acted as a within-subject factor. Planned contrasts for positive affect included comparing the PP condition against NP, PN and NN. In addition, when the ANOVA was significant, post hoc pairwise comparisons were carried out and statistical significance was adjusted for multiple testing according to Bonferroni. Omega squared was used to calculate effect sizes for repeated-measures ANOVA, and r was used for planned contrasts considering 0.1 as a small effect, 0.3 as medium, and 0.5 as large [20]. Analyses were conducted using SPSS version 26 (IBM Corp, Armonk, NY, USA).

## Results

### Task difficulty

On average, task difficulty was rated between “a little” and “moderately” in all conditions. The conditions involving the display of a smile (PN and PP) were rated as slightly harder, compared to the NN and NP (M_PN_ = 2.63; SD_PN_ = 1.35 and M_PP_ = 2.63; SD_PP_ = 1.50; compared to M_NN_ = 2.57; SD_NN_ = 1.70 and M_NP_ = 2.57; SD_NP_ = 1.74). However, repeated-measures ANOVA indicated that there was no main effect of condition on task difficulty (F (3,87) = 0.02; *p* = 0.99); thus, all tasks were similar in difficulty. We therefore proceeded with analysis as planned.

### Effects of facial and bodily emotional expressions on affect ratings

Table 2 shows the non-transformed means and standard deviations for self-reported positive and negative affect before and after the positive film clip was watched under each condition. The difference between the two scores was used as a measure of the affective experience.

**Table 2:** Experimental data, showing change in positive and negative affect in each experimental condition

Condition	Positive affect			Negative affect		
	Before positive film clip	After positive film clip	Difference	Before positive film clip	After positive film clip	Difference
	M (SD)	M (SD)	M (SD)	M (SD)	M (SD)	M (SD)
Neutral face + Neutral posture (NN)	16.07 (4.50)	21.57 (8.65)	5.50 (6.91)	20.97 (7.90)	10.67 (1.77)	−10.30 (8.03)
Neutral face + Positive posture (NP)	18.47 (5.61)	20.80 (9.57)	2.33 (7.05)	20.13 (8.73)	10.70 (1.24)	−9.43 (8.13)
Positive face + Neutral posture (PN)	17.93 (6.70)	20.50 (9.42)	2.57 (7.05)	19.97 (8.18)	10.57 (1.17)	−9.40 (7.37)
Positive face + Positive posture (PP)	15.23 (4.96)	21.53 (8.46)	6.30 (7.15)	17.60 (6.22)	10.80 (2.16)	−6.80 (5.08)

Table shows untransformed data

*M* mean, *SD* standard deviation

For positive affect, Mauchly’s test indicated that the assumption of sphericity was not violated (χ^2^ (5) = 8.18; *p* = 0.15). Results indicated that the changes in positive affect during the positive film clip were significantly affected by the different facial expressions and bodily postures (F (3,87) = 5.54; *p* < 0.01; ω^2^ = 0.03). Planned contrasts indicated that participants reported larger increases in positive affect during the PP condition compared to NP (F (1,29) = 7.89; *p* = 0.01; r = 0.46) and PN conditions (F (1,29) = 8.81; *p* = 0.01; r = 0.48), but not compared to NN (F (1,29) = 0.44; *p* = 0.51; r = 0.12). Post hoc pairwise comparisons adjusted for multiple testing using Bonferroni indicated that participants reported larger increases in positive affect during the PP condition, compared to PN (*p* = 0.04) and NP (*p* = 0.05), even though the latter was only a trend level. There were no differences between PP and NN conditions (*p* = 1.00). Other pairwise comparisons were not significant.

For negative affect, the assumption of sphericity was not violated (x^2^ (5) = 9.67; *p* = 0.09). Results indicated that the changes in negative affect during the positive film clip were not significantly affected by the different facial expressions and bodily postures (F (3,87) = 1.98; *p* = 0.12; ω^2^ = 0.01).

## Discussion

The study found that when participants adopted a positive facial expression and positive bodily posture, positive change in mood was significantly higher than when participants held a neutral face and a positive bodily posture or a positive face and a neutral bodily posture. Interestingly, there was no significant difference in positive mood change between the positive face and positive body condition and the neutral face and neutral body condition. This finding was unexpected and warrants further exploration.

Our findings lend some support to the notion that feedback is not limited to the face, suggesting that both face and body should be considered together when studying feedback effects to accurately reflect the emotional experience. Previous studies may well have underestimated feedback effects by considering either the face or the body separately.

The findings also suggest that incongruence between face and body is associated with smaller feedback effects. There is evidence that congruent facial and bodily expressions of emotions evoke stronger neural and physiological responses than incongruent displays [21]. It might be the case that displaying incongruent facial and bodily expressions provided mixed signals resulting in reduced feedback.

This pilot study explored a new design for manipulating both face and body simultaneously, which is important in furthering our understanding of how facial and bodily feedback work together in the real world. The study was carefully designed to overcome challenges faced in previous studies. For instance, the study employed a convincing cover story to do with art interpretation. This is known to be important in reducing demand characteristics [6]. The study also included important validity checks, wherein coders blind to the study design reviewed video footage and judged whether or not participants adhered to instructions. Furthermore, the study was designed with computerised instructions so as to deliberately minimise experimenter interaction. This is because of the potential for demand characteristics and experimenter expectancy effects to influence findings.

As part of running this pilot study, a number of methodological challenges were encountered which are relevant to the development of future studies involving both facial and bodily feedback.

Firstly, there was a substantial amount of non-compliance with the experimental task instructions, resulting in the exclusion of 16 participants’ data, limiting power. The reasons for this non-compliance are unclear. Instructions were tested in a prepilot study to ensure comprehension, and task difficulty was rated by participants as between “a little difficult” and “moderately difficult”. This substantial non-compliance points to the need for future studies to also conduct validity checks to ensure task instructions are followed adequately.

One potential explanation for the high non-compliance rate is that the videos used in this study were too long. Participants were asked to hold postures and facial expressions for durations of up to 2 min 53 s. There was particular non-compliance when participants were asked to hold a smile. Whilst participants did not report this condition to be more difficult than the others, it is likely that it would be effortful for participants to maintain this for over two minutes. Future researchers considering a similar design may wish to consider shorter clips or static images to reduce burden on participants [22].

It may also have been difficult for participants to modify their face and body simultaneously, whilst paying attention to the video clips. Future researchers may wish to consider non-intrusive environmental adaptations that may help to facilitate compliance. For instance, selecting chairs with adjustable backs for this study may have made it easier for participants to manipulate their posture.

Furthermore, compliance may have been aided by having the experimenter demonstrate the experimental conditions and having the participant practice ahead of beginning the study. As aforementioned, the choice to minimise interaction with the experimenter was deliberate in order to limit demand characteristics and experimenter expectancy effects. However, it is possible that a live demonstration of the study task by the experimenter may have aided compliance during the study. This could have been especially helpful given that 9 participants were excluded for not following instructions at all, indicating that they might not have had a full understanding of the study procedure from the computerized instructions alone.

A further challenge was in distinguishing between a neutral and a positive posture. This is because one would expect a neutral posture to be fairly similar to an upright posture. In this study, a slumped posture was selected as the neutral posture. However, it could be argued that this posture may be more negative than neutral, as it resembled negative postures from previous research [23]. It would be useful to explore further whether or not the idea of an emotionally ‘neutral’ posture is valid.

Future research should also include a mixed sample, as the present study sample only included females. It would be useful to replicate the study including males to ascertain whether the findings replicate.

## Conclusion

This pilot study explored a new design for manipulating both face and body simultaneously in order to measure the effects on positive modulation. Whilst the findings are difficult to interpret, there are a number of methodological suggestions for future studies of this kind in order to further our understanding of how the face and body work together to influence the emotional experience.
